# Supplementary material for: Examining isokinetic knee peak torque and time to peak torque as predictors of vertical jump height in division I men’s basketball players
Source: PLoS One. 2025 Apr 1;20(4):e0303701. doi: 10.1371/journal.pone.0303701 (PMC11961005; doi:10.1371/journal.pone.0303701)
Supplement: Attachment — Submitted filename: Response to Reviewers.docx [file pone.0303701.s001.docx]

**Response to Reviewers**

Dear Reviewers,

Below are your concerns addressed:

1. **Why did you decide to test from 90-0 degrees?**

Thank you for the comment. Range of motion limitations were set based on prior protocol and studies. We are not aware of any literature that would suggest that this many influence test results in regard to reps but would be happy to include any literature that does suggest that it may be a limitation.

2. **Name the Model of the Dynamometer**

This is addressed in the methods section. It is a Biodex Dynamometer.

3. **Do you think 30 seconds rest between speeds is enough?**

Thank you for the comment. We consider a 30 second break between speeds to be sufficient. Prior studies have concluded that there was no significant difference in knee extension or knee flexion peak torque when comparing work to rest ratios. We are not aware of any literature that would suggest that it isn’t but would be happy to include any literature that does suggest it isn’t enough and mention it in a limitations section.

4. **How come you decided to use an average and not peak torque values?**

Thank you for the comment. We consider the average value to be more stable of a variable than peak torque as the average value is based on all of the repetitions rather than just one repetition for the peak torque variable.

5. **Even more, you have obviously calculated average from both legs which is odd and provide**

**any reference for that. As isokinetic strength testing is my area of expertise I must say that you should use peak torque (not average) and dominant side (in basketball one used to push off in two-step).**

Thank you for your expert comment, we appreciate your expertise. Please see above our rational for using the average peak torque. The task we utilized in this study was a two-legged countermovement jump rather than a running vertical jump. Given this was a two-legged task, we consider the variables included in the statistical analysis to be appropriate for the manuscript.

6. **Also, I think that you are lacking jump without arm swing, as many studies have confirmed that arm swing may significantly influence the height of the jump and this in turns may explain why you didn’t find significant correlations with knee extensor strength. OK I see that you were not using force platform but Vertec - I hope this is in limitations**

Thank you for the comment. We only used the Vertec for the jump height which requires the use of the hands/arms. We will mention this in the limitations section with the follow language – “The current study allowed the subjects to use their arms/hands during the jumps which is necessary based on the used of the Vertec. This could have affected the ability to find correlations between jump height and knee extensor strength.”

7. **Your body weight normalized values are actually in %. Add % or change the decimal point. You are mixing anglosaxon and metric system. Check Plos instructions about units of measure. You are repeating some information from the Table in the text. Don’t do so.**

Thank you for your comment. The data presented in Tables 2 and 3 are calculated as a ratio of peak torque normalized to body weight and are presented as such. All units are updated to be correctly in the metric system (torque in Nm, weight as kg, and time in ms). Vertical jump was originally reported in inches and has been corrected to centimeters.

8. **Your linear regression model is prone to bias. Have you been doing multiple regression approach as well? Adding peak torque instead of average may significantly influence correlations as well as whole model.**

Thank you for the comment. Please see previous comments/response to this variable. We have included a statement in our limitations section.

9. **Can you explain in more details this assumption “It was hypothesized that time to peak torque at the highest °/ speed of 300 s would have the strongest correlation with VJ performance.” It is maximal strength that matters for VJ, and that one is best measured at low concentric velocities. If you wanted time include test power instead of time to peak torque**

Thank you for the comment. We created this hypothesis because the speed of 300 degrees/second likely is closer to the angular velocity of the actual movement.

10. **In the discussion section you are talking about the peak torque, but instead you were actually using average - see above.**

Thank you for the comment. We have updated the language in the discussion to reflect “average peak torque” as the variable of discussion.

11. **For a study showing really low R2 albeit significant I would tune down the suggestion in regard to that finding.**

Thank you for the comment.

Thank you for your review.
