# Supplementary material for: Examining isokinetic knee peak torque and time to peak torque as predictors of vertical jump height in division I men’s basketball players
Source: PLoS One. 2025 Apr 1;20(4):e0303701. doi: 10.1371/journal.pone.0303701 (PMC11961005; doi:10.1371/journal.pone.0303701)
Supplement: Attachment — Submitted filename: Response to Reviewers_2.docx [file pone.0303701.s002.docx]

**Response to Reviewers**

Dear Reviewers,

Below are your concerns addressed:

1. **The use of Vertec is a limitation.**

Thank you for the comment. The Vertec has been shown to be a valid and reliable measure for lower extremity testing. Use of the Vertec was based on prior protocol and studies. We are not aware of any significant limitations of Vertec that may influence test results but would be happy to include any literature that does suggest that it may be a limitation.

2. **The protocol used could (should) have ALSO considered 2 VJs that are part of Bosco's protocol: squat jump and countermovement jump. The work was left with almost no limitations, and the findings would undoubtedly have a more significant scientific impact. However, taking into account the rationale behind the modelling, it can be seen that the objective is focused, specifically, on using equipment without technological limitations that may be available at training locations. Thus, performance in VJ with vortex can, at any time, be interpreted in light of this work. However, it is essential to emphasise that the results of this study are only valid for these conditions. However, the use of the methodology stated is also an advantage due to the standardisation of the VJ movement, but because it resembles the VJ most performed by athletes in the studied modality. The question is: Which studies use the same methodology (BIODEX vs VERTEC)? The work would be significantly improved if the reference bibliography (mainly) focused on protocols with the same studied vertical jump (Vertec).**

Thank you for the comment, we appreciate your expertise. We considered the choice of a two-legged countermovement jump and variables included in the statistical analysis appropriate for the manuscript and this study population. We considered all reference bibliography appropriate for the discussion and relevant for comparison.

*Specific comments*

1. **Title: Suggestion: Examining Isokinetic Knee Peak Torque and Time to Peak Torque as Predictors of Vertical Jump Height using Vertec apparatus in Division I Men’s Basketball Players**

Thank you for your suggestion. We believe the current title is appropriate for the contents of the paper.

2. **Discussion**

   **L178-180: “Overall, the results of this study did not support this hypothesis, as none of the predictor variables showed a significant correlation with VJ height.” // No. The results support the hypothesis, i.e., time to peak torque at the highest speed of 300 °/s would have the strongest correlation with VJ performance. Review this sentence to clarify.**

Thank you for the comment. Our hypothesis was specific to the predictor variables; therefore, the results did not support this original hypothesis. We believe the following lines clarify that the regression analysis showed significance.

**L197-198: “This experiment failed to find a significant correlation between peak torque and VJ height, and this may be explained by the role of the hip joint during the VJ.” // You could also reference the VJ assessment methodology (i.e., using VERTEC, which is not a standard vertical jump test like the SJ, CMJ or ABK).**

Thank you for the suggestion. We addressed limitations in methodology in terms of the use of arm swing and generalizability of the subjects. As addressed above, we utilized VERTEC based on prior protocol and studies which showed reliability of its use.

**L2002: “semimembranosus and rectus femoris” // The muscles semitendinosus and (long head of) biceps femoris (booth from the posterior region) are missing (they are also biarticular muscles – hip and knee).**
Thank you for the comment, we will add these additional muscles into the sentence to properly reflect the biarticular muscles.

**3. Practical applications
This section is a mix of discussion and practical applications. I believe that to improve this section considerably, it can (should) be reformulated (i.e., present an objective and direct text in which the reasoning - and respective references - would only be considered in the “discussion” section).**

Thank you for your comments. We wrote this section such that the object is the “focus on rapid generation of torque in addition to traditional strengthening,” followed up with respective references in plyometrics, Olympic weightlifting, and male soccer players.

Thank you for your review.
